# Cell Connections by Tunneling Nanotubes: Effects of Mitochondrial Trafficking on Target Cell Metabolism, Homeostasis, and Response to Therapy

**DOI:** 10.1155/2017/6917941

**Published:** 2017-06-04

**Authors:** Marie-Luce Vignais, Andrés Caicedo, Jean-Marc Brondello, Christian Jorgensen

**Affiliations:** ^1^IRMB, INSERM, Montpellier University, Montpellier, France; ^2^Escuela de Medicina, Colegio de Ciencias de la Salud (COCSA), Universidad San Francisco de Quito (USFQ), Quito, Ecuador; ^3^Instituto de Microbiología, Colegio de Ciencias Biológicas y Ambientales, Universidad San Francisco de Quito (USFQ), Quito, Ecuador; ^4^Department of Therapeutic Immuno-Rheumatology, Lapeyronie University Hospital, Montpellier, France

## Abstract

Intercellular communications play a major role in tissue homeostasis and responses to external cues. Novel structures for this communication have recently been described. These tunneling nanotubes (TNTs) consist of thin-extended membrane protrusions that connect cells together. TNTs allow the cell-to-cell transfer of various cellular components, including proteins, RNAs, viruses, and organelles, such as mitochondria. Mesenchymal stem cells (MSCs) are both naturally present and recruited to many different tissues where their interaction with resident cells via secreted factors has been largely documented. Their immunosuppressive and repairing capacities constitute the basis for many current clinical trials. MSCs recruited to the tumor microenvironment also play an important role in tumor progression and resistance to therapy. MSCs are now the focus of intense scrutiny due to their capacity to form TNTs and transfer mitochondria to target cells, either in normal physiological or in pathological conditions, leading to changes in cell energy metabolism and functions, as described in this review.

## 1. Introduction: TNTs, What Are They? How Were They Discovered?

Cell communication is essential for tissue homeostasis, specific cell functions, and response to external cues. Indeed, during development and self-repair, tissues constantly need to adapt to changing biological conditions in order to reach physiological homeostasis. For this, their constituting cells constantly interact with target cells that reside in their close vicinity or alternatively, they can reach out to cells much further away, without necessarily involving the close-by surrounding cells. This cell-to-cell communication can be achieved by various processes including diffusible factors like cytokines and chemokines, secreted microvesicles, or direct passage through gap junctions. Long-distance diffusible factors can target different cell types, depending on the expression, by these cells, of the relevant receptors.

**Figure 1:**
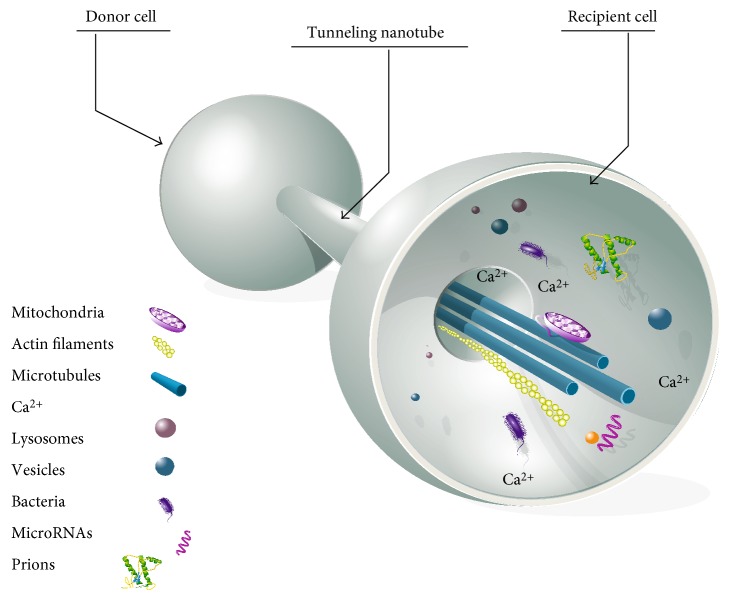
Tunneling nanotube (TNT). Tunneling nanotubes can connect many different cells together, using cytoskeleton actin microfilaments, microtubules, or both. TNTs allow the trafficking, from donor to recipient cells, of cargoes including organelles, proteins, miRNAs, and ions.

Another impressive means of communication cells devised to allow long-distance cell-to-cell contacts are the formation of tunneling nanotubes (TNTs) between these cells, as initially reported in the rat pheochromocytoma- (PC12-) derived cells and in immune cells [1, 2]. These are long tubular structures, with diameters between 50 and 1500 nm, that can span several tens to hundreds of microns, connecting two cells together [3]. In a characteristic manner, in 2D cultures, TNTs are not tethered to the extracellular matrix, rather floating in the culture medium. Microscopy imaging, either of live or of fixed cultures, proved very useful to characterize these cellular structures [3–10]. The tunneling nanotubes allow a continuity in plasma membrane and cytoplasm between the connecting cells, thus allowing trafficking of a number of cellular components from one cell to the other. This trafficking can rely on cytoskeleton fibers, of either actin, microtubules, or both (Figure 1 and [3]).

In the past few years, a number of studies reported this capacity of cells, from an ever increasing number of cell types, to connect to one another. Interestingly, these TNTs also allow the trafficking of a number of different cargos between the connected cells, therefore increasing the combinatorial complexity of these cell-to-cell connections and their biological outcome, as summarized in Table 1. In this review, we provide a general overview of what is currently known about tunneling nanotubes, the cells involved, the cargoes transported within TNTs, and the regulation of these processes. We further focus on the specific capacity of mesenchymal stem cells (MSCs) to connect to target cells through such TNT structures and to transfer mitochondria to the targeted cells, emphasizing the modifications in the energetic metabolism and the biological functions the MSC mitochondria generate in these cells. Due to space constraints, we do apologize in advance for articles we could not cite.

## 2. How Are TNTs Formed? What Are the TNT-Connected Cell Partners?

### 2.1. Cell Types Involved in TNT Connections (Table 1)

Cells involved in connections through nanotubes can be of the same or of different types. Many cell types appear endowed with the capacity to form TNTs with one another. TNTs were observed among rat pheochromocytoma (PC12) cells [8, 11], renal proximal tubular epithelial cells (RPTEC) [12], rat kidney cells [13], and retinal pigment epithelial cells [14]. Tunneling nanotube formation was also reported between endothelial progenitor cells and endothelial cells [15], between endothelial progenitor cells and cardiac myocytes [16], and between immature hippocampal neurons and astrocytes [17]. Noteworthily, even though tunneling nanotubes have been largely described in human and murine systems, they were also reported in bacteria, connecting *B. subtilis* cells together and *B. subtilis* with the distantly related *E. coli* [18], in Drosophila where they contribute to niche-germline stem cell signaling [19] and in the zebrafish during gastrulation [20].

Cells of the immune system, notably macrophages, dendritic cells (DCs), NK, and B cells, extensively use TNTs to communicate [6, 21–27]. Shortly after the discovery of TNTs in PC12 cells, these structures were also identified between DCs and monocytes [28]. The transfer of antigenic information from migratory DCs to lymph node-residing DCs through TNTs was recently shown to be critical for the induction of immune responses [24]. TNT formation was also described in neural CAD cells (mouse cell line of catecholaminergic origin) and from bone marrow-derived dendritic cells to primary neurons [6, 25, 26].

As it will be further described below, mesenchymal stem cells (MSCs) actively use TNTs to deliver cargos to renal tubular cells [29], cardiomyocytes [30], bronchial epithelial cells [31, 32], macrophages [33], endothelial cells [34], and breast cancer cells [35, 36]. Reciprocally, MSCs can receive cargos from TNT-connected cells as in the case of human vascular smooth muscle cells (VSMCs) [9].

Formation of TNTs has been observed for a number of cancer cells, either connecting cancer cells together or connecting cancer cells with normal stromal cells, notably mesenchymal stem cells (MSCs). TNT formation was described in a diversity of different cancer cell types, including malignant mesothelial cells [37–40], colon carcinoma cells [41], MCF7 and MDA-MB-231 breast cancers, SKOV3 and OVCAR3 ovarian cancers [36], K7M2 murine osteosarcoma cells [37], laryngeal squamous cell carcinoma (LSCC) [42], HeLa cells [43, 44], astrocytoma cells [45], and bladder cancer cells [46]. TNT formation was also observed between normal and cancer cells, including between nonmalignant IOSE human ovarian epithelial cells and SKOV3 ovarian cancer cells [37], between stromal MC3T3 murine osteoblast cells and K7M2 osteosarcoma cells [37], and between HeLa cells and fibroblasts, in both directions [44]. A number of the connections between normal stromal cells and cancer cells were found to involve MSCs, as for instance in the following studies involving MDA-MB-231 breast cancer cells [35], B cell precursor acute lymphoblastic leukemia (ALL) cells [47], and acute myeloid leukemia cells [48].

The nanotubes formed between these different cell types do share some features, notably a continuity in cell membrane and cytoplasm between the connected cells, allowing the trafficking of biological cargos. However, with the accumulation of new TNT-related data, it appears that these structures will have properties, concerning the connecting modes, cargos transported, cytoskeleton-based molecular motors, and biological outcome that will underline the specificity of each cell system.

### 2.2. Cargos Transported within the TNTs

#### 2.2.1. Mitochondria

Organelles such as mitochondria have now been described as trafficking entities in the tunneling nanotubes connecting many different cells types including renal proximal tubular epithelial cells (RPTEC) [12], astrocytes [49], astrocytomas [45], endothelial cells [50], neuronal CAD cells [25], laryngeal squamous cell carcinoma (LSCC) [42], and monocyte-derived macrophages [33]. This mitochondria transfer was also observed between endothelial and cancer cells [36], endothelial progenitor cells and cardiac myocytes [16], and from healthy to damaged (UV-treated) PC12 cells [8].

**Figure 2:**
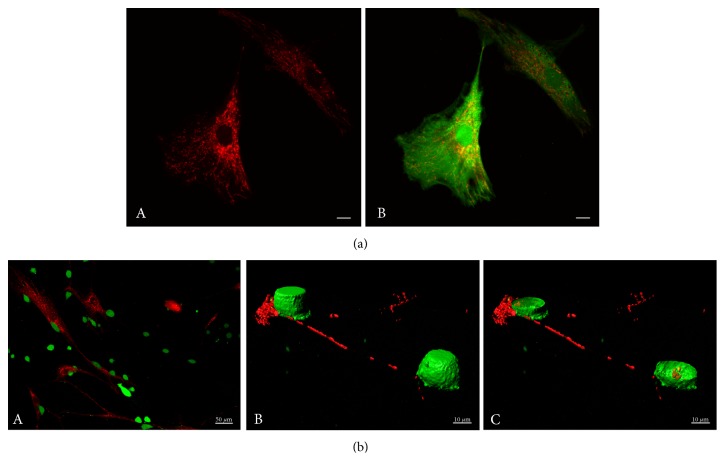
Mitochondrial trafficking from MSCs to MDA-MB-231 breast cancer cells. (a) MSC mitochondrial network. MSCs were labeled by MitoTracker Deep Red FM and Green CellTracker CMFDA. Scale bars, 10 *μ*m. (b) Transfer of MSC mitochondria to MDA-MB-231 cells. Coculture (24 h) of human MSCs (MitoTracker Red CMXRos prestained) and MDA-MB-231 cells (Green CellTracker CMFDA prestained). (A) 2D view of the coculture, (B, C) 3D reconstruction of the cells from stacks of confocal images with the cell isosurface view (B), and *xy* plane section (C) (Imaris). Scales, (A) 50 *μ*m, (B, C) 10 *μ*m.

MSCs were shown to share mitochondria through a TNT-mediated process with number of target cells. These target cells include cardiomyocytes [30], endothelial cells [34], pulmonary alveolar epithelial cells [31, 32], renal tubular cells [29], macrophages [33], and acute myeloid leukemia cells [48] as well as breast cancer cells (Figure 2) [35, 36], leading to modifications of the functional properties of these cells. Interestingly, in this latter case, the MSC mitochondria transfer was observed to be of a higher extent for the leukemic CD34^+^ myeloblasts than for the normal mononuclear CD34^+^ cells [48]. Conversely, when MSCs were in coculture with vascular smooth muscle cells, the TNT-mediated mitochondrial trafficking resulted in the acquisition of the VSMC mitochondria by the MSCs [9].

On a technical point of view, detection of the transfer of mitochondria from donor to target cells is often performed based on the imaging of mitochondria prelabeled with fluorescently dyes such as MitoTrackers as shown in numerous reports, including [35, 51]. Detection of the transferred mitochondria can also be performed by genetically labeling the donor cells by the lentiviral-mediated expression of a GFP fusion protein with the mitochondrial cytochrome c oxidase subunit VIII (LV-Mito-GFP) [52]. Alternatively, heterologous systems, that is, human/mouse or human/rabbit, allow the use of antibodies like the monoclonal anti-human mitochondria antibody (MTC02) that specifically recognizes human mitochondria, but not mitochondria of mouse or rabbit origin, and can, thus, enable to discriminate between the transferred human mitochondria and the endogenous [35, 53]. Mitochondria contain their own mitochondrial DNA (mtDNA) that is transferred, as expected, concomitantly with the trafficking mitochondria. Because mitochondria from unrelated donors harbor mtDNA with specific SNPs, these punctual nucleotide differences in the mtDNA can also be used as a tool to distinguish and quantify the mtDNA of the transferred mitochondria in respects to the endogenous ones [35, 51].

As it will be discussed later in the review, this mitochondrial trafficking leads to notable effects in the target cells as mitochondria are involved in multiple cellular functions including the biosynthesis of ATP, through the electron transport chain, or that of lipids and amino acids. In addition, mitochondria are now recognized as signaling entities that can induce cell events such as autophagy and apoptosis.

#### 2.2.2. Other Cargoes

Among organelles, lysosomes were also found to be transferred between progenitor and senescent endothelial cells and this transfer [15]. The TNTs formed between CAD cells (mouse neuronal cell line of catecholaminergic origin) were shown to allow the transfer of lysosomes and also of prions (PrP^Sc^) from the infected to noninfected cells [25, 54]. A similar TNT-mediated PrP^Sc^ transfer was observed from bone marrow-derived dendritic cells to primary neurons providing a possible route for prions to the brain [25]. PrP^Sc^ was recently suggested to transfer, as well, from infected murine astrocytes to primary cerebellar granule neurons through tunneling nanotubes [55]. The protein Tau can also be transferred as a fibrillar protein between neurons, therefore possibly contributing to Tau pathologies [7].

Viruses also display the capacity to be transmitted through TNTs. This was shown for HIV, between infected T cells and noninfected T cells, thus eliminating the need for the infected cells to release a fully mature HIV virus in order to infect the neighboring cells [56, 57]. In addition, HIV-containing TNTs were described to be formed by infected macrophages, connecting them to other macrophages [58] and between noninfected and HIV-infected DCs [24].

TNTs also constitute a route for the transfer of microRNAs between cells, as shown for miR-19a among K7M2 murine osteosarcoma cells, [37]. The transport of miRNA was observed between cancer cells and the normal cells of the surrounding tumor microenvironment, as demonstrated between the K7M2 osteosarcoma and the stromal MC3T3 osteoblast cells and between the human SKOV3 ovarian cancer cells and nonmalignant IOSE ovarian epithelial cells [37]. In addition, the presence of lipid droplets was also detected in the TNTs connecting human microvascular endothelial cells (HMEC-1). The number of these lipid droplets was found to increase under angiogenic conditions (VEGF) and in response to arachidonic acid [50]. Ions like calcium (Ca^2+^) can also be transmitted between TNT-connected cells, as shown from DCs to monocytes [23], for SH-SY5Y neuroblastoma and HEK cell lines [59], for ARPE-19 human retinal pigment epithelial cells [14], and for astrocytomas [45].

### 2.3. TNT Formation Mechanisms and Molecular Motors

Two major processes have been proposed for the formation of TNTs. Cells can extend filopodia-like protrusions that, in contact with target cells, can undergo plasma membrane fusions. Alternatively, cells that were initially in close contact with one another can move apart, remaining bound by the extending tunneling nanotube structure. Several proteins have now been identified for their role in nanotube formation, for the functional connection between the two interacting cells, and for the cargo trafficking within the connecting TNTs.

The role of connexin 43 (Cx43) gap junction marker has been documented for different cell systems. In the murine model of LPS-induced acute lung injury, gap junctions between the instillated murine bone marrow stromal cells (BMSCs) and the pulmonary alveolar epithelial cells depended on the expression of Cx43 by both cell types and occurred at sites of high-Cx43 expression. Cx43 was therefore proposed as essential for BMSC attachment to the alveolar cells, leading to the generation of TNTs between these cells [32]. A high expression of the Cx43 was also observed at the neuronal contact site of the TNTs formed between hippocampal neurons and astrocytes [17]. Expression of Cx43, and not that of other connexins, was reported for TNTs formed between astrocytoma cells [45]. Finally, among the different connexins expressed by human laryngeal squamous cell carcinoma (LSCC), Cx43 was the one required for gap junction and TNT formation among LSCCs [42].

The role of M-Sec/TNFaip2 and the exocyst complex has also been put forward in different studies [5, 56]. Interestingly, M-Sec-induced TNTs were found to contain actin filaments, but not microtubules [5]. Other factors, like LST1 (leukocyte-specific transcript 1), were found to contribute to the formation of nanotubes through the exocyst complex, by recruiting the small GTPase RalA to the plasma membrane and promoting its interaction with the exocyst complex [60]. In HeLa cells, TNT formation involved the action of the GTPase Rab8 [44].

Another small GTPase, Cdc42, was found to play a dual role in TNT formation. Cdc42 was demonstrated to play a role in the TNT elongation process in the Raw264.7 macrophage cell [5] and to favor protein trafficking from oncogene- or DNA damage-induced senescent cells to NK cells [10]. However, it was the GTPase RalA, and not Cdc42, that was shown to be important for LST1-induced nanotubes in HeLa cells [60]. On the other hand, Cdc42 (together with IRSp53) and VASP (vasodilator-stimulated phosphoprotein) were found to inhibit TNT formation in neuronal CAD cells [61].

The mitochondrial trafficking within these TNTs can rely on the Rho GTPase Miro1 (also called RhoT1/2), as shown for the transfer of mitochondria from mesenchymal stem cells to damaged alveolar epithelial cells in mouse models of airway injury [31]. Miro1 was also found to play a key role and be responsible for the differences in mitochondrial transfer efficacies observed between iPSC-MSCs and adult BM-MSCs [62]. As a matter of fact, mitochondrial transportation has been thoroughly studied in polarized cells, such as neurons, where mitochondrial production of ATP at distant sites from the cell body is crucial to meet local energy demands. The Rho GTPase Miro1 was shown to connect to the kinesin-1 molecular motor through the Milton adaptor protein (also called TRAK1/2 and OIP106/98), enabling mitochondrial transport along microtubules [63].

Other cytoskeleton motors can allow the transport of small molecules and organelles within the cells. In addition to the kinesin motor, the cytoplasmic dynein also moves along microtubules while, on the other hand, the family of myosins are actin-based cytoskeleton motors [64]. The molecular motor myosin-X (Myo10) was also proposed as a key regulator of tunneling nanotube formation in murine neuronal (CAD) cells, increasing the occurrence of TNTs and the transfer of vesicles in these TNTs [6].

Depending both on the types of cells connected and cargos transported, it is likely that TNTs will rely on different types of cytoskeletons, that is, microfilaments and/or microtubules, and therefore on different cytoskeleton motors to support the trafficking of these cargos. For instance, the protein Tau was reported to associate with both microtubules and the actin network and to contribute to the formation of TNTs, bridging neurons together [7]. On the other hand, mitochondria were found to traffic along microtubules in PC12 cell-connecting TNTs [8]. Interestingly, cytochalasin D, latrunculins A, and B, that are potent inhibitors of actin polymerization, were shown for instance to inhibit TNT formation between MSCs and vascular smooth muscle cells [9] or between senescent cells and NK cells [10].

Altogether, the diversity of factors involved in the formation of the TNTs and of the cargoes trafficking within these TNTs points to the complexity of the whole process of TNT-mediated cell-to-cell communication. New paradigms will be needed to allow to predict which cargoes might be transferred, using what type of cytoskeletal motor, for any given couple of cell types.

### 2.4. Regulation of TNT Formation

The formation of TNTs, as tested in 2D in vitro cultures, was observed to be controlled by several factors including serum and glucose concentrations, viral infection, or exposure to therapeutic agents, as detailed further below. Beyond the fact that this information is important to design experimental settings and collect in vitro data on TNTs, it also gives clues about how TNT formation might be regulated in vivo, by nutrient supply, infection, or therapy, and thus contribute to our understanding of the holistic organism responses.

In vitro, low-serum (2.5% FBS) and high-glucose concentrations (50 mM) were found to stimulate TNT formation, as observed between murine K7M2 osteosarcoma cells and MC3T3 osteoblast cells [37]. Low-serum, hyperglycemic, acidic growth medium was also used to stimulate both the formation of TNTs and the mitochondrial trafficking between malignant or between normal mesothelial cells [39]. Rat hippocampal astrocytes and neurons, as well as HEK293 kidney cells, produced more TNTs at low-serum concentration and upon H_2_0_2_ stimulation [49]. Concerning the effects of glucose concentrations, it is worth mentioning that, for neuronal mitochondrial trafficking, high-glucose concentrations were shown to diminish mitochondrial motility, by a mechanism involving Milton and its O-GlcNAcylation by the O-GlcNAc transferase (OGT) [65]. Given the apparent similarities between the processes of mitochondrial trafficking in neurons and in TNTs, a possible role of glucose-dependent OGT activation for mitochondrial trafficking within TNTs might be worth checking. Albeit the effects of high-glucose concentrations reported above, it is also worth noting that, in other cell systems, it is the glucose deprivation that was found to enhance the TNT-mediated mitochondrial transfer, as observed from MSCs to endothelial cells [34].

Cellular stress caused, for instance, by HIV infection in human macrophages was demonstrated to increase the number (but not the length) of TNTs formed by these macrophages towards other macrophages, in correlation with viral replication [58]. Chemotherapeutic agents have also been found to influence the occurrence of TNT formation and cargo trafficking. Zeocin is a DNA-intercalating agent related to bleomycin that induces cell death by causing double-strand breaks. Zeocin treatment of renal proximal tubular epithelial cells (RPTEC) was found to increase up to 10-fold (for 400 ng/ml Zeocin) the number of TNTs formed by these cells [12]. Cytarabine (ARA), a nucleoside analog used as a chemotherapeutic agent to treat AML, was found to increase physical interactions between AML cells and bone marrow-derived MSCs and mitochondrial incorporation by the AML cells [48]. This effect on mitochondrial uptake by AML cells was also observed following treatment with the topoisomerase II inhibitor etoposide and the anthracycline doxorubicin, but not for the microtubule-disrupting agent vincristine [48]. Finally, other mechanisms were also linked to TNT formation. They could involve cell activation by CD40L, a member of the TNF family, shown to activate DC TNTs [24]. Besides, the enhanced expression of p53 was shown to be important for TNT formation by MG63 osteosarcoma cell TNTs [49] while dispensable for TNT formation in other cell types as shown for PC12 cells, OCI-AML3 (acute myeloid leukemia) cells, human osteosarcoma cell line SAOS-2 (p53-null), and murine bone marrow-derived MSCs [66].

## 3. Mesenchymal Stem Cells: Characterization and Functional Properties

### 3.1. MSC Properties

Mesenchymal stem cells (MSCs) are characterized by their multilineage differentiation capacity, notably into osteocytes, adipocytes, and chondrocytes [67–71]. They also express specific cell surface markers that include CD105 (endoglin) [72], CD73 [73], CD90, CD19 [74], CD79 [75], CD14 [76], CD11b [77], and HLA-DR [70], but not the hematopoietic markers CD45 [78]. Additional MSC surface markers, like Stro-1, SSEA-4, CD271, and CD146, have also been proposed [79]. MSCs are found in nearly all tissues. Outside from the bone marrow-derived mesenchymal stem cells (BM-MSCs), MSCs have now been isolated from other tissues including the human placenta, umbilical cord, spleen, and adipose tissue-derived mesenchymal stem cells (AT-MSCs) [80, 81]. These MSCs share common properties but also exhibit differences in the expressed cell markers, in their differentiation potential and phenotypes [82–84]. As an example, both BM-MSCs and AT-MSCs show a high expression of CD271 and of the Stro-1 marker compared to MSCs from other tissues, while CD146 (or MCMAM (melanoma cell adhesion molecule)) appears specific to BM-MSCs [79]. Interestingly, CD146 is also a marker of pericytes, proposed to give rise to MSCs following blood vessel damage or inflammation [85–87]. It is worthwhile noting that, even when isolated from a single tissue, MSCs can form a heterogeneous population with diverse differentiation and immune regulatory capacities [79, 82, 88].

### 3.2. Role of MSCs in Immune Suppression

MSCs are attracted and activated by cytokines such as IFN-*γ*, TNF-*α*, IL-6, IL-8, IL-1, and TGF-*β* that are present at high concentrations in the inflammatory environment [89–91]. MSC exposure to this inflammatory environment contributes to their tissue repair and immunosuppressive properties. Once at the inflammation site, MSCs prevent cellular destruction and damage to surrounding tissues [90–92]. MSC immuno-suppression is mediated by the secretion of soluble factors like indoleamine 2,3-dioxygenase (IDO), IL-10, TSG-6 (TNF-*α*-stimulated gene/protein 6), prostaglandin E2 (PGE2), TGF-*β*-1, inducible nitric oxide synthase (iNOS) and human leukocyte antigen (HLA-G) [93–97]. Interestingly, different mechanisms were proposed for the murine and human MSC immunoregulatory properties. For example, murine and human MSCs, respectively, produce inducible nitric oxidase synthase (iNOS) and indoleamine 2,3-dioxygenase (IDO), both iNOS and IDO contributing to the immunosuppressive functions of MSCs [98].

### 3.3. Role of MSCs in the Tumor Microenvironment

The tumor microenvironment is known to play an important role in tumor progression, metastasis, and resistance to therapy [99, 100]. Mesenchymal stem cells are recruited to the tumor microenvironment where they have the capacity to modify the growth and metastatic potential of the cancer cells [88-89, 99, 101–104]. The recruitment of MSCs to the tumor microenvironment depends on a number of cytokines and chemokines secreted by the tumor cells [105, 106] which can give rise to mutual cross talks between MSCs and cancer cells [102, 107]. The MSCs present in the tumor microenvironment also display the capacity to modify the response of nearby cancer cells to therapeutic agents [99] as exemplified for cisplatin [108] and paclitaxel [109]. Interestingly, multiple reports now establish that the response of the cancer cells to therapy is closely linked to the metabolic reprogramming of these cells [110, 111].

## 4. Capacity of MSCs to Connect to Target Cells via Nanotubes and Biological Outcomes

### 4.1. TNT Connections and Mitochondria Transfer between MSCs and Target Cells

MSCs interact with other cells, reprograming their function through the secretion of small molecules like growth factors, chemokines, cytokines, and molecular mediators (bioactive lipids, nucleotides, among others). The human mesenchymal stem cells (MSCs) have been shown to display the ability to connect to target cells through tunneling nanotubes and to transfer the mitochondria through these TNTs. Prockop laboratory observed for the first time that functional mitochondria could be transferred between MSCs to tumor cells [112]. These target cells now include cardiomyocytes, endothelial cells, pulmonary alveolar epithelial cells, renal tubular cells, and cancer cells, leading to modifications of the functional properties of these cells [8, 29–32, 34–36, 48, 113].

### 4.2. Other Mechanisms for MSCs to Transfer Mitochondria

Mitochondria can also be transported from MSCs to the other cells by microvesicles (MVs). MSC mitochondria can be taken by arrestin domain-containing protein 1-mediated microvesicles (ARMMs) that range from 0.1 to 1 *μ*m in diameter; therefore, fitting mitochondria whose average size is of the order of 0.5 *μ*m. These microvesicles were shown to be engulfed by macrophages, resulting in increased macrophage mitochondrial bioenergetics [114]. This MV-dependent mitochondria transfer between MSCs and macrophages was thus proposed to be beneficial for both macrophages and MSCs, as it also decreased the MSC load of depolarized mitochondria [114]. Mitochondria were also reported to be released by astrocytes as mitochondria-containing particles, in a CD38-dependent process, and recaptured by neurons [115].

### 4.3. Mitochondria, Isolated beforehand from Cells, Can Be Transferred to Target Cells

Mitochondria, in an isolated form, can also be internalized by cells, notably cardiomyocytes [35, 51, 53, 116, 117]. This process has been proposed to depend on macropinocytosis [116, 117]. The quantitative transfer of isolated mitochondria to target cells by the technique of MitoCeption, based on this capacity of mitochondria to be internalized by living cells, was shown to be a tool of choice to determine the effects of the transferred mitochondria in the target cells [35]. Other techniques to transfer the target cells of mitochondria, isolated beforehand, include formation of transmitochondrial cybrids [118] and the use of photothermal nanoblades [119] as well as the direct cell injection of mitochondria, as performed in oocytes [120–122].

### 4.4. Mitochondrial TNT Transfer Observed In Vitro Also Occurs In Vivo

Islam and colleagues demonstrated the transfer of mitochondria in vivo from MSCs to pulmonary alveolar epithelial cells in a murine model of lipopolysaccharide- (LPS-) induced acute lung injury [32]. After their instillation in the lungs of mice with LPS-injured alveoli, MSCs were found to form gap junctions with the injured lung epithelial cells. This resulted in the transfer of MSC mitochondria to these cells and to the regeneration of the affected alveoli. Furthermore, this study pinpointed the role of connexin 43-containing gap junctions for the efficient in vivo transfer of the MSC mitochondria [32]. Likewise, Ahmad and colleagues used a mouse model of rotenone-induced acute lung injury that enabled them to demonstrate the role of MSCs and their transferred mitochondria in the rescue of the injured bronchial epithelial cells [31]. This in vivo model helped establish the role of the Rho-GTPase Miro1 in the mitochondrial intercellular trafficking. The TNT-mediated in vivo transfer of MSC mitochondria was also observed in a rat model of cigarette smoke-induced chronic obstructive pulmonary disease (COPD). In this model, human MSCs were demonstrated to protect from alveolar destruction through mitochondrial transfer to the rat airway epithelial cells, moreover, with a higher efficiency for the iPSC-MSCs than for the BM-MSCs [123].

Lung alveolar macrophages were also shown to acquire MSC mitochondria, which lead to an enhancement of their phagocytic activity and, thus, contributed to the MSC antimicrobial effect in a murine model of *E. coli*-induced pneumonia [33]. The in vivo mitochondrial transfer from the endogenous murine bone marrow stromal cells was demonstrated as well in a NSG immunodeficient murine model of xenograft of human AML cells, on the basis of the expression of the mtDNA-encoded murine Co2 RNA [48].

The transfer to target cells of mitochondria, isolated beforehand from cells, was also demonstrated in vivo in a rabbit model of regional ischemia [53]. The injection of autologous mitochondria (~10^7^ mitochondria) at the site of ischemia lead to their internalization within 8 hours of their administration and resulted, among other phenotypes, in reduced apoptosis and infarct size, as detected 4 weeks later [53].

## 5. Functional Outcome of MSC Mitochondrial Transfer to Target Cells

### 5.1. Effect on Metabolism

The transfer of MSC mitochondria to A549 *ρ*° adenocarcinoma cells, following their coculture, led to the recovery of the mitochondrial function in these cells, including O_2_ consumption [112]. The metabolic effects of the acquired mitochondria were demonstrated in a number of studies in vitro. The coculture of MSCs and endothelial cells (HUVEC) was found to lead to an increase of HUVEC basal and maximal oxygen consumption, while glycolysis and lactate production were concomitantly reduced [34]. A similar effect (increase in OXPHOS, decrease in glycolysis) was observed for MDA-MB-231 breast cancer cells that had acquired human MSC mitochondria. These data were obtained using the technique of MitoCeption that allows a quantitative transfer of mitochondria, isolated beforehand from the MSCs. It showed a dose-response effect of the MSC mitochondria on the cancer cell metabolism [35]. In addition, the concentrations of both the endogenous mitochondrial DNA and the produced ATP were increased [35]. Similar increases in ATP concentrations were also reported in acute myeloid cells following their coculture with bone marrow-derived MSCs (both human and murine) [48]. In addition, a beautiful study of Islam and collaborators demonstrated in vivo, in a LPS-induced lung injury model and using single-cell ATP determination, that ATP concentrations increased in the alveoli cells that had received MSC mitochondria and, thereafter, spread to adjacent alveoli [32].

### 5.2. Effect on Cell Function and Survival in Response to Therapy

The acquisition of human vascular smooth muscle cell (VSMC) mitochondria by human MSCs resulted in the increase of MSC proliferation rate [9]. Such an enhancement of the capacity of cellular proliferation, as well as invasion, was also observed for the MDA-MB-231 breast cancer cells after acquisition of human MSC mitochondria [35]. Acquisition of MSC mitochondria induced cardiomyocyte reprogramming to a progenitor state, characterized notably by markers such as GATA-4, myocyte enhancer factor 2C, and Nkx2.5 [30].

In a rabbit ischemia model, the injection of autologous mitochondria at the site of ischemia resulted in their internalization by cardiomyocytes and in increased cell survival [53]. In the two mouse models of acute lung injury following LPS [32] or rotenone [31] treatments, airway instillation of MSCs and MSC mitochondrial transfer to alveoli resulted in alveoli functional rescue and mice survival. MCF7 breast cancer cells with acquired endothelial cell mitochondria were reported to display increased resistance to doxorubicin [36]. Acquisition of exogenous mitochondria by AML cell allowed them to maintain their overall mitochondrial membrane potential and increased their survival rate in response to ARA treatment [48]. This increased survival was also observed for the leukemia-initiating cells (LICs) that play a major role in AML relapse [48].

## 6. Conclusion and Perspectives

Tunneling nanotubes appear henceforth to constitute a widespread means of communication between cells that can lay close-by or far apart. This communication process is used by many cell types, allowing the trafficking of many different cargoes between these cells. This TNT-mediated cell-to-cell exchange can contribute to the cell homeostasis, to the spontaneous tissue repair, to the spreading of pathologies, and to the resistance to therapies.

As detailed in this review, mesenchymal stem cells are particularly prone to establish these TNT connections with target cells. Numerous studies reported and characterized effects that the mitochondrial trafficking in these TNTs can have on the target cells be at the metabolic or functional levels. On a therapeutic point of view, at a first glance, these effects can be beneficial, when they lead for instance to tissue repair, but also detrimental, when they contribute to acquired resistance to therapy. Obviously, further work will be necessary to find the tools to enhance the first while hindering the second. The fact that mitochondria can be transferred spontaneously between cells or from preparation of mitochondria, isolated beforehand, will obviously open new paradigms for the available options to treat patients.

## Figures and Tables

**Table 1:** 

Authors	TNT donor cells	TNT receiver cells	Transported cargoes	References
Onfelt et al. (2004)	Human NK cells	Human EBV-transformed human B cells	GFP-tagged cell surface class I MHC	[2]
	Human macrophages	Same cells		
	Human EBV-transformed human B cells	Same cells		
	Murine J774 macrophages	Same cells		

Rustom et al. (2004)	Rat pheochromocytoma PC12	Same cells	Microvesicles	[1]
	Human embryonic kidney (HEK)	Same cells	Organelles	
	Normal rat kidney (NRK)	Same cells		

Castro et al. (2005)	Colon carcinoma cell line SW620	Same cells	ND	[41]

Koyanagi et al. (2005)	Human endothelial progenitor (EPC)	Neonatal rat cardiomyocytes (CM)	Mitochondria	[16]

Watkins et al. (2005)	Human dendritic cells	Same cells and THP-1 cells	Calcium flux	[23]
	Human THP-1 monocytes	Same cells	Major histocompatibility proteins (MHC class I)	

Chinnery et al. (2008)	Murine MHC class II dendritic cells	Same cells	ND	[21]

Gurke et al. (2008)	Normal rat kidney cells (NRK)	Same cells	Endocytic organelles	[13]

Onfelt et al. (2006)	Human macrophages	Same cells	Bacteria	[28]
			Mitochondria	
			Vesicles (endosomes, lysosomes)	

Sowinski et al. (2008)	Jurkat T cells	Same cells and primary T cells	HIV viral particles	[57]
	Primary T cells	Same cells		

Bukoreshtliev et al. (2009)	PC12 cells	PC12 cells	Intracellular organelle transfer	[11]

Eugenin et al. (2009)	Human macrophages	Same cells	HIV viral particles	[58]

Plotnikov et al. (2010)	Human mesenchymal multipotent stromal cells (MMSC)	Rat renal tubular cells (RTC)	Mitochondria	[29]

Acquistapace et al. (2011)	Human mesenchymal stem cells (MSCs)	Cardiomyocytes	Mitochondria and intracellular material	[30]

Domhan et al. (2011)	Human proximal tubular epithelial cells (RPTEC)	Same cells	Microvesicles	[12]

Wang et al. (2011)	Rat hippocampal astrocytes	Same cells and rat hippocampal neurons	Endoplasmic reticulum	[49]
	Rat hippocampal neurons	Same cells and rat hippocampal astrocytes	Mitochondria	
			Golgi fragments	
			Endosomes	
			Amyloid *β*	

Yasuda et al. (2011)	Human umbilical vein endothelial cells (HUVEC)	Stressed HUVEC	Lysosomes	[15]
			Mitochondria	

Islam et al. (2012)	Murine MSCs	Murine alveoli	Mitochondria	[32]

Lou et al. (2012)	Human primary cancer cells	Same cells	Mitochondria	[39]
	Human mesothelial lines (MSTO-211H, VAMT, H-Meso)	Same cells		

Schiller et al. 2012	HeLa	Same cells	Transmembrane HLA-A2-EGFP protein	[43]

Vallabhaneni et al. (2012)	Human MSCs	Human vascular smooth muscle cells (VSMCs)	Mitochondria	[9]

Wittig et al. (2012)	Human retinal pigment epithelial (ARP-19) cells	Same cells		[14]

Costanzo et al. (2013)	CAD cells	Same cells and with transfected CADs	Htt aggregates	[26]
	Primary cerebellar granule neurons (CGNs)	Same cells and with transfected CGNs		

Pasquier et al. (2013)	Human mesenchymal stem cells (MSCs)	Same cells and ovarian and cancer cell lines	Mitochondria	[36]
	Human endothelial cells (HECs)	Same cells and ovarian and cancer cell lines		
	Human ovarian cancer cells (SKOV3, OVCAR3, HTB-161)	Same cells		
	Human breast cancer cells (MDA-MB231 and MCF7)	Same cells		

Rainy et al. (2013)	Human B cells	Human T cells	Plasma membrane-associatedproteins (H-Ras)	[27]

Ady et al. (2014)	VAMT (sarcomatoid mesothelioma cell line)	Same cells	ND	[38]
	H2052 (mesothelioma cell line)	Same cells		
	MSTO-211H (derived from mesothelioma patient)	Same cells		
	Met5A (immortalized mesothelioma cell line)	Same cells		

Ahmad et al. (2014)	Murine MSCs	Murine lung epithelial cells	Mitochondria	[31]

Liu et al. (2014)	Human MSCs	Human umbilical vein endothelial cell (HUVEC)	Mitochondria	[34]

Thayanithy et al. (2014)	Murine osteosarcoma K7M2 cells	Same cells and MC3T3 murine osteoblasts	MicroRNAs (miR-199a)	[37]
	SKOV3 ovarian cancer cells	Nonmalignant ovarian epithelial cells		

Thayanithy et al. (2014)	Human biphasic mesothelioma MSTO-211H cells	Same cells	Exosomes from other cells	[40]

Biran et al. (2015)	Oncogene or DNA damage-induced senescent cells	NK cells	Proteins	[10]

Burtey et al. (2015)	HeLa	NRK fibroblasts	Tf-R (transferrin receptor), endosomes	[44]

Caicedo et al. (2015)	Human mesenchymal stem cells (MSCs)	Human breast cancer cell line MDA-MB-231	Mitochondria	[35]

Polak et al. (2015)	Bidirectional: human MSCs to human acute lymphoblastic leukemia cells (BCP-ALL cell line) Bidirectional: human MSCs to human B cell precursor of Leukemia Nalm6 (B-Other) Bidirectional: human MSCs to human acute lymphoblastic leukemia cells (TEL-AML1)		ND	[47]

Wang and Gerdes (2015)	PC12 cells (−/+ultraviolet light treatment)	PC12 cells (−/+ultraviolet light treatment)	Mitochondria	[8]

Zhu et al. (2015)	CAD neuronal cells	Same cells	Prions	[54]
			Lysosomes	
			Early endosomes	

Hashimoto et al. (2016)	Monocyte-derived macrophages	Same cells	HIV-1	[56]

Hayakawa et al. (2016)	Astrocytes	Neurons	Mitochondria	[115]

Jackson et al. (2016)	Human MSCs	Human monocyte-derived macrophages	Mitochondria	[33]
		Murine alveolar macrophages		

Lu et al. (2016)	Bladder cancer cells	Same cells	Mitochondria	[46]

Moschoi et al. (2016)	BM-MSCs	Acute myeloid leukemia cells	Mitochondria	[48]

Tardivel et al. (2016)	Neurons	Neurons	Tau protein	[7]

Victoria et al. (2016)	Astrocytes	Neurons	Prions	[55]

Zhang et al. (2016)	iPSC-MSCs and BM-MSCs	Cardiomyocytes	Mitochondria	[62]

## Abbreviations

ALL: Acute lymphoblastic leukemia
AML: Acute myeloid leukemia
ARA: Cytarabine or cytosine arabinoside
AT-MSC: Adipose tissue-derived mesenchymal stem cells
BM-MSC: Bone marrow-derived mesenchymal stem cells
CM: Cardiomyocytes
COPD: Chronic obstructive pulmonary disease
Cx43: Connexin 43
DC: Dendritic cells
DOXO: Doxorubicin
EPC: Endothelial progenitor cells
ETO: Etoposide
FBS: Fetal bovine serum
GTPase: Guanosine triphosphatase
HIV: Human immunodeficiency virus
HMEC-1: Human microvascular endothelial cells
ICAM-1: Intercellular adhesion molecule 1
IDO: Indoleamine 2,3-dioxygenase
iNOS: Inducible nitric oxide synthase
IRSp53: Insulin receptor substrate of 53 kDa
LIC: Leukemia-initiating cell
LPS: Lipopolysaccharide
LSCC: Laryngeal squamous cell carcinoma
LST1: Leukocyte-specific transcript 1
MCMAM: Melanoma cell adhesion molecule
MSCs: Mesenchymal stem cells
Mt-Co2: Mitochondrially encoded cytochrome c oxidase II
mtDNA: Mitochondrial DNA
MVs: Microvesicles
Myo10: Myosin-X
NK: Natural killer
NOD: Nonobese diabetic
NRK: Normal rat kidney
NSG: NOD scid gamma
OGT: O-GlcNAc transferase
PC12: Rat pheochromocytoma
PGE2: Prostaglandin E2
RPTEC: Renal proximal tubular epithelial cells
SNP: Single-nucleotide polymorphism
TNFaip2: Tumor necrosis factor, alpha-induced protein 2
TNT: Tunneling nanotube
TSG-6: TNF-*α*-stimulated gene/protein 6
VASP: Vasodilator-stimulated phosphoprotein
VEGF: Vascular endothelial growth factor
VINCRI: Vincristine
VSMC: Vascular smooth muscle cell.
